# Regulatory networks in retinal ischemia-reperfusion injury

**DOI:** 10.1186/s12863-015-0201-4

**Published:** 2015-04-24

**Authors:** Kalina Andreeva, Maha M Soliman, Nigel GF Cooper

**Affiliations:** Department of Anatomical Science and Neurobiology, University of Louisville, School of Medicine, 500 S. Preston Street, Louisville, KY 40292 USA

**Keywords:** miRNAs, Transcription factors, Regulatory networks, Retinal ischemia, Rat

## Abstract

**Background:**

Retinal function is ordered by interactions between transcriptional and posttranscriptional regulators at the molecular level. These regulators include transcription factors (TFs) and posttranscriptional factors such as microRNAs (miRs). Some studies propose that miRs predominantly target the TFs rather than other types of protein coding genes and such studies suggest a possible interconnection of these two regulators in co-regulatory networks.

**Results:**

Our lab has generated mRNA and miRNA microarray expression data to investigate time-dependent changes in gene expression, following induction of ischemia-reperfusion (IR) injury in the rat retina. Data from different reperfusion time points following retinal IR-injury were analyzed. Paired expression data for miRNA-target gene (TG), TF-TG, miRNA-TF were used to identify regulatory loop motifs whose expressions were altered by the IR injury paradigm. These loops were subsequently integrated into larger regulatory networks and biological functions were assayed. Systematic analyses of the networks have provided new insights into retinal gene regulation in the early and late periods of IR. We found both overlapping and unique patterns of molecular expression at the two time points. These patterns can be defined by their characteristic molecular motifs as well as their associated biological processes. We highlighted the regulatory elements of miRs and TFs associated with biological processes in the early and late phases of ischemia-reperfusion injury.

**Conclusions:**

The etiology of retinal ischemia-reperfusion injury is orchestrated by complex and still not well understood gene networks. This work represents the first large network analysis to integrate miRNA and mRNA expression profiles in context of retinal ischemia. It is likely that an appreciation of such regulatory networks will have prognostic potential. In addition, the computational framework described in this study can be used to construct miRNA-TF interactive systems networks for various diseases/disorders of the retina and other tissues.

**Electronic supplementary material:**

The online version of this article (doi:10.1186/s12863-015-0201-4) contains supplementary material, which is available to authorized users.

## Background

Retinal ischemia is a consequence of restrained blood flow that causes severe imbalance between the supply and the demand of nutrients and oxygen resulting in neuronal damage and impaired retinal function [1]. Immediate reperfusion attenuates the retinal damage, however, it is accompanied by mechanisms such as excessive reactive oxygen species (ROS) generation, low nitric oxide, and inflammation, and might accelerate neuronal cell death [2-4]. Retinal ischemia-reperfusion (IR) injury is associated with a wide range of conditions [5-9] that can culminate in blindness due to relatively ineffective treatment [10]. Detailed understanding of the molecular events following ischemia-reperfusion induced retinal damage would facilitate development of relevant treatments.

It is widely acknowledged that complex diseases and/or disorders, including those resulting in altered vision, are more likely linked to groups of genes, gene modules or gene pathways than to any single gene [11,12]. The transcriptional regulation of genes is mediated in part by transcription factors (TFs), while their post-transcriptional regulation is mediated in part by small non coding RNAs, a prominent class of which are microRNAs (miRs) [13]. Despite the different levels of regulation, both transcriptional and post-transcriptional regulatory interactions are not isolated from each other, but interact to execute complex regulatory programs which, in turn, modulate cellular functions [14,15]. Cellular and tissue functions rely on well-coordinated molecular interactions between genes, TFs and miRs, all integrated within regulatory networks [16,17]. The networks are fairly complex, and consist of a variety of patterns of interaction. For example, one possible pattern consists of a miRNA and a TF that coordinate one another and which also co-regulate a common gene [15] or gene-transcript. Since there is no general nomenclature to name this pattern, at this time, we have used the term “closed loop-motif” throughout this manuscript. The “closed loop” infers an interaction between all 3 elements of the loop. The “motif” part of the terminology infers a special relationship of the closed loop to some context driven activity within gene networks. In these closed loop-motifs, the TF, miRs and genes/transcripts can be viewed as nodes whereas the regulatory influences between them are seen as connecting lines or edges [18-20]. Since the loop motifs are highly interconnected within a regulatory network, the altered expressions of context-driven genes might also influence the expression of genes from neighboring loop-motifs. Emerging evidence indicates that loop-motifs which contain disease-driven differentially expressed molecular components (genes, miRs or TFs) are linked to different aspects of the etiology and/or expression of diseases and/or disorders [21,22].

In recent years extensive efforts have been focused on modeling of regulatory networks combining TFs and miRs [15,23-25]. The majority of these early studies focused on the development of algorithms or tools but did not address the biological context of the networks [25-28]. Furthermore, the construction of regulatory networks related to particular disorders is still in the very early stages of development. However, advances in the construction of such networks is essential and will eventually contribute to the identification of better drug targets and biomarkers for monitoring and controlling the progression of more complex disorders, such as glaucoma, ophthalmic artery occlusion and other retinopathies associated with retinal ischemia.

The goal of this study is to construct regulatory networks associated with early and late reperfusion time points following retinal ischemia and to capture transient changes in the regulatory networks.

## Methods

### Ischemia-Reperfusion injury (IR-injury) related mRNAs, TFs and miRNA

Microarray data were obtained and analyzed for miRNA and mRNA transcript levels for reperfusion times of 0 h, 24 h and 7d after an initial 1 h period of ischemia as previously described [29]. A total of 36 animals were used for the mRNA microarray study. Sham control and IR injured animal groups contained 18 rats per group. Each of the sham and IR injury related groups were divided into 3 sub-groups of 6 animals based on the 3 time points used for this study (0 h, 24 h, 7d). A total of 60 animals were used for the miRNA microarray study. Sham control and IR injured animal groups contained 30 rats per group. Each of the sham and IR injury related groups were divided into 5 sub-groups of 6 animals based on the 5 time points used for this study (0 h, 2 h, 24 h, 48 h, 7d). The treatment and care of all animals used in this study were approved by the University of Louisville Institutional Animal Care and Use Committee (IACUC) and were performed in accordance with the ARVO Statement for the Use and Care of Animals in Ophthalmic and Vision Research. The mRNA and miRNA datasets are deposited into the Gene Expression Omnibus (GEO) data repository (GSE43671 and GSE61072), where the information about the data normalization is available. In brief, the raw data files for the mRNA array (.txt) were imported into GeneSpring (GX 11.1) for normalization and analyses. GeneSpring generates an average value from the six animal/samples for each gene. Data were transformed to bring any negative values or values less than 0.01 to 0.01 and then log2-transformed. Normalization was performed using a per-chip 75 percentile method that normalizes each chip on its 75 percentile, allowing comparison among chips. Then a per-gene on median normalization was performed, which normalized the expression of every gene on its median among the samples. We retained a total of 23897 transcripts for further statistical analysis.

The raw data files with total 350 miRNAs extracted from Agilent Feature extraction software were further processed and analyzed by GeneSpring GX10.0 software. The raw data were at first normalized with the following conditions and then filtered by the flag using GeneSpring GX10.0 software. The normalization included log2 transformation, per chip normalization to 75% quantile and dropped per gene normalization to median. We retained the 219 normalized miRNAs for the further statistical analysis.

The expression values in IR-injured retinas were compared with those in sham control animals. Data reduction was performed on the datasets such that mRNAs and miRNAs, whose expressions were altered two or more times (absolute fold-change ≥ 2, and corrected P-value ≤ 0.05) in injured versus sham control animals were used for further analyses. The numbers of the identified mRNAs, miRs and TFs that are differentially expressed at 0 h, 24 h and 7d post-IR stages are shown in Table 1.

**Table 1:** **Differentially expressed mRNAs, TFs and miRs at 0 h, 24 h, and 7d post-IR periods, filtered by fold change ≥ 2 and p-Value ≤ 0.05**

**Differentially expressed molecular components**	**0 h**	**24 h**	**7d**
**mRNA**	31	919	678
**TFs**	2	45	39
**miRs**	10	66	63

There were a total of 43 molecular components with altered expression at the 0 h reperfusion time point after a period of 1 h ischemia. This number increased significantly to 1030 by 24 h and then decreased to 780 by 7d in the post ischemic periods. There were fewer TFs than miRs or mRNAs across all time points, and there were fewer miRs than mRNAs across all time points, so that the number of TFs < miRs < mRNAs.

### Inference of closed loop-motifs

**Figure 1:**
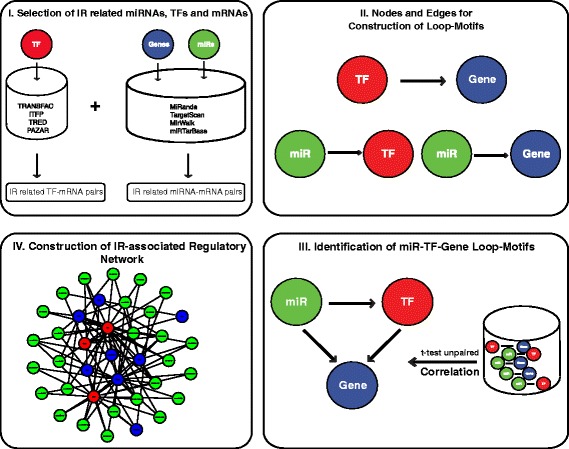
Overview of the workflow for construction of IR-injury-associated regulatory networks. In the first step **(I)**, we collected IR-related miRNAs, TFs and mRNAs from the experimental mRNA- and miRNA-arrays produced in our laboratory. These represent the altered expression values of the 3 elements detected at 3 different time points during ischemia-reperfusion injury. We then constructed TF-mRNA pairs, miR-mRNA pairs, and miR-TF pairs with the aid of external databases and/or software **(II)**. The paired constructs were used to build novel closed loop-motifs consisting of 3 nodes relationally interconnected by 3 edges **(III)**. The motifs were further integrated into IR-injury associated regulatory networks that consist of interconnected loop-motifs **(IV)**. Green filled-circles denote miRs, red filled-circles denote TFs and blue filled-circles denote target genes (transcripts).

The workflow for construction of IR-injury associated regulatory networks is diagrammed (Figure 1). To identify likely miRNA-mRNA pairs, miRNA target genes were collected from four publicly available databases: MiRanda [30,31] (August 2010 release), TargetScan [32] (release 6.2), miRWALK [33] (March 2011 release) and miRTarBase [34] (release 4.5). We considered the unified set of targets instead of the intersection of targets from these databases. Reportedly, the former provides more likely targets [35,36]. Identified target genes for all significant differentially expressed miRs in our datasets were submitted as miR-gene pairs to our own local database if the gene targets were also in our datasets of significant differentially expressed mRNAs (Figure 1.I).

To identify TFs in the rat genome as well as TF-target gene pairs, we used three publicly available databases (ITFP [37], PAZAR [38,39] and TRED [40,41]) as well as the commercial database TRANSFAC [42] (professional release 2014). Additionally, the Match Analysis tool [43] associated with TRANSFAC was used to investigate the promoter regions of genes (5 kb upstream) to identify predicted TF-target gene pairs. To minimize false positive as well as false negative relationships, only pairs of transcription factors and genes with the highest matrix score (0.8) were collected. Genes unknown to TRANSFAC were re-analyzed with the aid of Match using either different aliases (gene symbol or RefSeq ID) or through use of the promoter sequence of the gene as found with the UCSC table browser [44].). We added connecting edges to the 3 types of pairs; TF-Gene; miR-TF and miR-Gene without regard to direction of interaction (Figure 1.II).

Subsequently, we constructed putative tripartite loops by attaching edges between the interactions previously paired. These tripartite loop-motifs contain 3 different molecular entities, mRNA, miR, TF (Figure 1.III). The loop-motifs are building blocks and these are then combined to form the larger regulatory networks (Figure 1.IV). Due to the complex nature of the different relationships that might exist in a regulatory network, we restricted our inference to loop-motifs where the miR targets a TF and both co-regulate the expression of a co-targeted gene. Other combinations were not considered. We obtained a total of 4218 loop-motifs for the 24 h post-IR period and 957 loop-motifs for the 7d time point. These data were further reduced since only loops with three significantly correlated edges were considered (see below).

### Functional analysis

To explore the functional role and the underlying biological processes associated with the loop-motifs from the 24 h and 7d post-IR periods, the mRNAs in the TF-mRNA and miRNA-mRNA pairs were subjected to enrichment analysis using DAVID (Database for Annotation, Visualization and Integrated Discovery) [45,46] and IPA (Ingenuity Pathway Analysis, IPA®,QIAGEN Redwood City, CA). The most enriched biological processes, associated p-values and enrichment scores are listed in Table 2.

**Table 2:** **Functional analysis of mRNAs within the miRNA-TF-TG closed loop-motifs at 24h and 7d**

**Biological process**	**Time point**	**Enrichment score**	**P-value**	**Closed loop-motifs**
Cell death**	Early (24 h)	≥2.79	≤1.7E-3	1047
Ion transport*		2.13	7.3E-5	367
Synaptic activity**		≥1.1	≤7.7E-1	285
Apoptosis*		2.95	1.0E-3	144
Caspase activity*		1.74	1.5E-2	39
		**Total loop-motifs 24h**		**1278**
Cell death**	Late (7d)	≥2.7	≤2.3E-3	316
Antigen presentation**		≥4.11	≤3.8E-9	113
Ion transport**		≥2.67	≤1.1E-4	100
Immune response *		8.1	5.8E-12	98
Inflammatory response*		11.41	2.6E-9	45
		**Total loop-motifs 7d**		**413**

*Biological processes based on analyses by DAVID.

**Biological processes based on combined analyses by DAVID and IPA. In the combined analyses we summed the genes assigned by DAVID and IPA to the same biological process. The enrichment scores and p-values were obtained from either DAVID or IPA based on which of them provided the lowest p-values and the highest enrichment scores.

The mRNAs in the TF-mRNA and miRNA-mRNA pairs were analyzed with the aid of DAVID and IPA to find the biological processes affected by the IR-injury. The top biological process terms were identified by combining results from DAVID and IPA. DAVID and IPA provided the enrichment scores and their associated p-values. The numbers of closed loop-motifs for each biological process were then based on the mRNAs associated with each process. Note: there are closed loop-motifs that are shared among the biological processes and this reflects their total numbers (see Figures 5 and 7). All loop-motifs are part of the larger regulatory networks seen in Figure 3.

### Evaluation of regulatory loops

In order to estimate the reliability of the individual loop motifs and to provide a statistically rigorous framework, we evaluated the closed loop-motifs by examining the association between their 3 elements using two methods of correlations including Pearson correlation (ρ) [47,48] and distance correlation (DC) [49,50]. The classical measure of dependence, the Pearson correlation coefficient, is an association measure sensitive mainly to linear dependency between variables and has been used previously for inferring regulatory networks [51]. For two variables, X and Y, their Pearson correlation coefficient is defined as the covariance of the two variables divided by the product of their standard deviations (σ):

$$ \rho \left(X,Y\right)=\frac{\mathrm{cov}\left(\mathrm{X},\mathrm{Y}\right)}{\upsigma \mathrm{X}\upsigma \mathrm{Y}} $$

Although the prevailing approach when inferring regulation relationships is to assume linear dependencies between bio-elements, it is possible for some elements to have nonlinear dependency. DC is a novel method for evaluating nonlinear dependency that has many appealing features when compared to Pearson. Unlike Pearson, DC scores zero if and only if variables are independent (a Pearson correlation of zero does not imply independence between variables). Since our miRNA array data were generated for 5 time points (0 h, 2 h, 24 h, 48 h and 7d) and mRNA for only 3 time points (0 h, 24 h and 7d) post-IR injury, we imputed mRNA expression data for two additional time points (2 h and 48 h) using the simple least square method [52-54]. This approach allowed us to calculate both, linear and nonlinear dependencies for all predicted miRNA-mRNA pairs at matching time points. Detailed results from both correlation methods could be found in Additional file 1.

Each inferred loop motif consists of three edges (miR-TF, miR-mRNA, and TF-mRNA), and each individual edge was tested for both linear and nonlinear dependency. The R packages Hmisc (http://cran.r-project.org/web/packages/Hmisc/index.html) and Energy (http://cran.r-project.org/web/packages/energy/index.html) were used to calculate ρ and DC values between elements of each loop motif. The significance of ρ and DC values were supported by an associated p-value. Only loops with all three significantly correlated edges (p-value ≤ 0.05) were considered for further analyses. The combined correlation analysis (ρ and DC) resulted in 2681 out of 4218 (63.6%) closed loop motifs associated with the 24 h post-IR period and 699 out of 957 (73%) closed loop-motifs in the 7d post-IR period (Table 3).

**Table 3:** **Number of closed loop-motifs and their molecular components for each of the three reperfusion time points (0 h, 24 h, and 7d) following 1 h of ischemia**

**Closed loop-motifs**	**0 h**	**24 h**	**7d**
Closed loop motifs	**0**	**2681**	**699**
**mRNA**	0	433	215
**TFs**	0	16	14
**miRNA**	0	53	34

Only loop-motifs, which contained 3 statistically significantly and correlated pairings (miR-TF, miR-mRNA, and TF-mRNA) were listed here (p-value ≤ 0.05). It is noteworthy that although there were 43 differentially expressed molecular components at 0 h, there were insufficient numbers of statistically significant and correlated pairings at this initial time point.

### Construction of regulatory networks

Significantly correlated closed loop-motifs identified in the previous step were integrated into regulatory networks associated with 24 h and 7d time points following retinal IR-injury. The Gephi open graph visualization platform [55] was used to develop graphic representations of the regulatory networks containing nodes, each consisting of either miR, TF, or TG and their interconnecting edges representing interactions between the nodes. We analyzed the topological structure of the networks to identify regulators (TFs and miRs) with major regulatory roles in 24 h and 7d post-IR-injury based on node degree. The node degree is defined as the number of directly connected neighbors of a node in a particular network. Nodes that have a high number of directly connected neighbors are thought to be important regulatory hubs within the regulatory network.

## Results

### Analysis of regulatory closed loop-motifs associated with IR-Injury

Initial analyses indicated that different numbers of mRNAs, TFs and miRs were present at the three different time points following the initial ischemic condition (Table 1). The lowest number of changes occurred at 0 h whereas the largest number occurred at 24 h. These changes were reflected in the number of closed loop motifs observed for each time point (Table 3). Thus, there were no closed loop motifs at the 0 h time point, and the maximum number was observed at 24 h. The absence of closed loop motifs at 0 h may indicate a lack of sensitivity or a lack of data at this time point. This is an area that may need further investigation.

**Figure 2:**
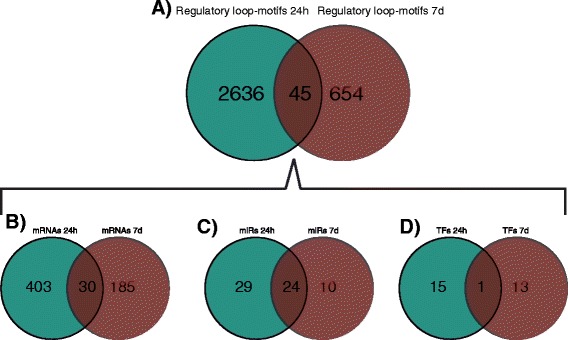
Unique and common loop-motifs and individual molecular loop-components related to early (24 h) and late (7d) stages post-IR injury. At 0 h, there were no significant closed loop-motives. **A**. Closed loop-motifs. **B**. Transcripts (mRNAs) representing target genes. **C**. microRNAs (miRs). **D**. transcription factors (TFs). There were a higher number of loop motifs at 24 h than at 7d. Moreover, there were more representations of mRNAs, miRs and TFs at 24 h compared with the 7d, whether they were unique or common. The pattern of node relationships for the molecular components is typical in that the numbers of TF < miRs < mRNAs.

In contrast, at the 24 h reperfusion time point, there were 433 mRNAs (47.1% from all differentially expressed mRNAs), 16 TFs (35.5% from all differentially expressed TFs) and 53 miRs (80.3% from all differentially expressed miRs) from which we were able to construct 2681 closed regulatory loop-motifs (Table 3). At the 7d reperfusion time point there were 215 mRNAs (31% from all differentially expressed mRNAs), 14 TFs (35.9% from all differentially expressed TFs) and 34 miRNAs (54% from all differentially expressed miRNAs) from which we were able to construct 699 closed regulatory loop-motifs (Table 3). Comparison of the motifs between time points revealed the presence of only 45 overlapping loop motifs. These common regulatory loop motifs involved 30 mRNA, 24 miRs and a single TF, which was Stat1 (Figure 2). These results indicate, for the most part, different regulatory motifs are linked to distinct ischemic-reperfusion time points which would likely have some prognostic value.

### Properties of time point specific regulatory networks associated with IR-Injury

**Figure 3:**
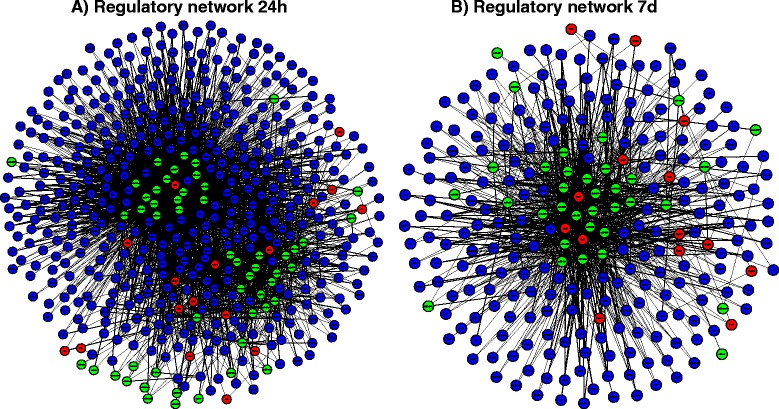
Highly interconnected networks of loop motifs related to early (24 h) and late (7d) post-IR injury periods. **A**. Regulatory network at 24 h consisting of 2681 closed loop-motifs, with only 45 in common with the 7d time point. **B**. Regulatory network at 7d consisting of 699 closed loop-motifs, with only 45 in common with the 24 h time point. Each motif is composed by a microRNA (miR, filled green circle) a transcription factor (TF, filled red circle) and a related protein-coding gene transcript (filled blue circle). Every node (miRNA, TF and TG) represents a differentially expressed molecular element with altered expression in retinal ischemia-reperfusion injury, when compared to sham control animals.

The regulatory network at the 24 h post-IR stage integrated 2681closed loops and consisted of 504 nodes and 3214 edges (Figure 3A), while the network at the 7d post-IR stage combined 699 closed loops and contained 263 nodes and 1032 edges (Figure 3B). Thus topological network analysis revealed higher connectivity at 24 h (3214 edges) compared to 7d (1032 edges).

To assess the overall contribution of the individual elements (miRs, mRNAs, TFs) within nodes to the expansiveness of the networks at each time point, the node degrees (or levels of connectivity) were calculated for each node. The top 10 mRNAs, TFs and miRs in both networks were ranked by their degrees and listed (Table 4).

**Table 4:** **Top ten transcription factors, mRNAs and miRs ranked by the number of their connections at the 2 time points 24 h and 7d post-IR injury**

**miR (24 h)**	**C**	**miR (7d)**	**C**	**mRNA (24 h)**	**C**	**mRNA (7d)**	**C**	**TF (24 h)**	**C**	**TF (7d)**	**C**
miR-495*	172	miR-873	55	Dhcr24	22	Igf1	21	Maf	389	Lef1	124
miR-214	170	miR-223	48	Map2	22	Prrx1	16	**Stat1**	165	**Stat1**	106
**miR-207**	143	miR-410	45	Hmox1	21	Xpr1	16	Creb1	104	Bcl6	98
miR-298	132	miR-185	45	Pcdha4	21	Dhcr24	14	Nptx1	29	Stat3	12
miR-466b	111	miR-291a-3p	44	Foxp1	21	Slc26a4	14	Tp53	28	Runx1	8
miR-206	104	**miR-495**	43	Samd12	20	Glce	13	Foxp1	21	Cebpb	8
miR-19a	102	miR-329	40	Kcnc1	19	Slc18a2	13	Isl1	18	Litaf	7
miR-221	95	**miR-207**	35	Ppp1r9a	19	Stat3	12	Nr2c2	15	Arida5a	6
miR-297	91	miR-138	31	Rictor	19	Fut4	12	Gnb1	12	Lgals3	4
miR-33	90	miR-539	28	Dclk1	19	Scarb2	12	Rnf138	7	Arpc1b	3

*Bold denotes the common molecular components between the early and late post-IR time points.

We have rank-ordered each of the 3 molecular components by their relative connectivity and used this as a marker of relative importance within networks at the 2 time points. Note that there are a few molecular components which are common to both time points. For example rno-miR-495 and rno-miR-207 are common between the 24 h and 7d time points, but they are rank-ordered differently between the 2 time points. In addition, *Stat1* is present and equally rank-ordered at both time points. C = number of connections.

Reportedly, the nodes that have a high degree of connectivity are known as hub nodes (or hubs) and play major roles in the regulatory networks. The top three rno-miRs at 24 h were rno-miR-495* (degree of 172), followed by rno-miR-214 (degree of 170) and rno-miR-207(degree of 143). In contrast at 7d, rno-miR-873 (degree of 55), rno-miR-223 (degree of 48) and miR-410 (degree of 45) had the highest degrees of connectivity. The top three TFs were *Maf* (degree of 389), *Stat1* (degree of 165) and *Creb1* (degree of 104) in the network associated with the 24 h time point and *Lef1* (degree of 124), *Stat1* (degree of 106) and *Bcl6* (degree of 98) in the regulatory network associated with the 7d post-IR. Of particular note, at both time points, with the exception of the top 3 TFs, most TFs had relatively low levels of connectivity (Table 4). We didn’t distinguish between in- and out-degree and we ranked the molecular components based on connectivity (the sum of in- and out-degree). However, further analyses showed that the ranking of the top 3 TFs would not change if we consider the out-degree only. The top 10 gene-transcripts, distinct for each of the 24 h and 7d time points, were moderately well connected at between 12 and 22 connections each.

The gene-transcripts in the regulatory loops in these networks were evaluated for their biological relevance with the aid of the Database for Annotation, Visualization and Integrated Discovery (DAVID) [45,46] and pathway analysis (Ingenuity Pathway Analysis, IPA®,QIAGEN Redwood City, CA) and the most enriched biological processes were listed (Table 2). The networks associated with the 24 h time point were significantly enriched for genes participating in cell death, apoptosis, caspase-activation, ion transport and synaptic activities. The networks associated with the 7d time point were significantly enriched for genes participating in inflammatory responses, immune responses, antigen presentation, ion transport and also cell death. Similarities and differences between the processes in each time point are discussed below.

### Sub-networks at 24 h post-IR period

**Figure 4:**
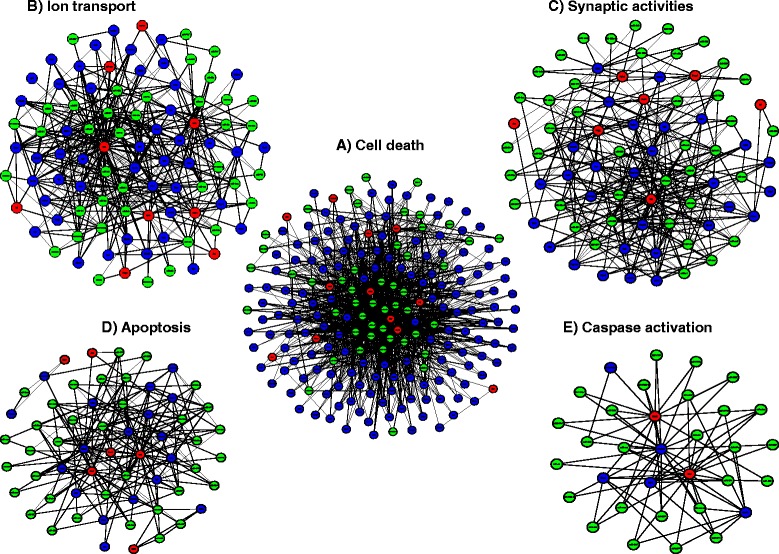
Sub-networks at 24 h associated with 5 particular cellular processes, all being a part of the larger regulatory network seen in Figure 3A. Each of the subnetworks contains multiple closed loop-motifs. **A**. Loop-motifs related to cell death. **B**. Loop-motifs related to ion transport. **C**. Loop-motifs related to synaptic activity. **D**. Loop-motifs related to apoptosis. **E**. Loop-motifs related to caspase activation. Green filled-circles denote miRs, red filled-circles denote TFs and blue-filled circles denote target gene-transcripts.

Within the large network of closed-loop motifs associated with the 24 h time point (Figure 3A) there are several prominent sub-networks (Figure 4A-E), which together represent approximately 50% of all regulatory loops detected. The numbers of statistically significant closed loop motifs for each of these smaller sub-networks are listed (Table 2). The global transcription factors *Maf*, *Creb1* and *Stat1* are the principal regulatory components in each of these sub-networks, and the target genes corresponded to the annotated biological functions. For example, potassium inwardly-rectifying channels (*Kcnj12, Kcnj3, Kcnj9*), voltage-gated potassium channel *Kcnc*1, the protein kinases (*Jak3, Prkca, Prkce*), the genes encoding solute carrier membrane transport proteins (*Slc12a2, Slc38a3, Slc4a8, Slc4a7, Slc4a10, Slc8a1)* and the voltage gated sodium channel *Scn2a1* were the represented target genes in the sub-network linked to ion transport (Figure 4B), while the glutamate receptors *Gria4* and *Grm5*, gamma-aminobutyric acid (GABA) B receptor *Gabbr2*, synaptotagmin I (*Syt1*) and inositol 1,4,5-trisphosphate receptor *Itpr1* were among the nodes in the sub-network associated with synaptic activities (Figure 4C). In contrast, the target genes from the sub-network associated with apoptosis (Figure 4D) were mostly shared with target genes in the caspase activation associated network (Figure 4E). This is to be expected since caspase activation is a hallmark of apoptosis.

**Figure 5:**
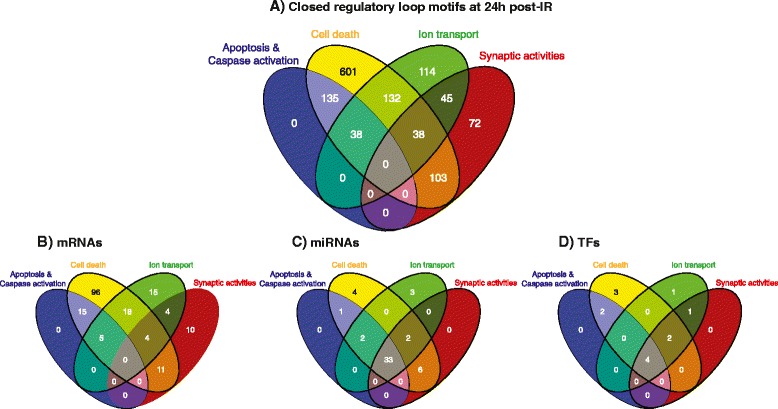
Venn diagrams representing the relative contribution of cellular processes and the numbers of their unique and overlapping loop motifs and molecular components at 24 h. **A**. The numbers of common and unique loops-motifs associated with 4 different cellular processes. **B**. The common and unique mRNAs associated with 4 different cellular processes. **C**. The common and unique miRNAs associated with 4 cellular processes. **D**. The common and unique TFs associated with 4 cellular processes. The 4 colored oval shapes represent different biological processes. Blue: apoptosis and caspase activation, yellow: cell death, green: ion transport, red: synaptic activity.

Since all of the biological processes identified in the 24 h regulatory network ultimately lead to cell death, they are likely to share regulatory motifs. We combined the sub-processes in 4 groups: cell death, synaptic activity, apoptosis and caspase (combines apoptosis and caspase activation) and ion transport. Their comparison is illustrated in Figure 5. Each of the processes shared regulatory loops with the cell death sub-network. For example, all the motifs associated with apoptosis and caspase-activation were shared with the motifs belonging to the cell death regulatory sub-network. A large number of motifs (208 out of 367) linked to ion transport were shared with the cell death sub-network. Another 141 motifs (out of 258) linked to synaptic activities were also shared with the cell death sub-network (Figure 5A). Differing numbers of motifs were shared between two or three of the regulatory sub-networks, indicating that all biological sub-processes at the 24 h time point are closely linked. We also explored the common and unique mRNAs, miRs and TFs between the biological sub-processes at 24 h post-IR (Figure 5 B, C and D). No common target mRNAs among all biological processes at the 24 h time point were identified. In contrast, a large number of miRs (33) but very few TFs (4) were common among the biological processes. Taken together, the results infer that a few TFs together with a small group of miRs coordinate the regulation of a large number of different sub-networks within larger composite networks thereby affecting regulation of different biological processes.

### Sub-networks at 7d post-IR period

**Figure 6:**
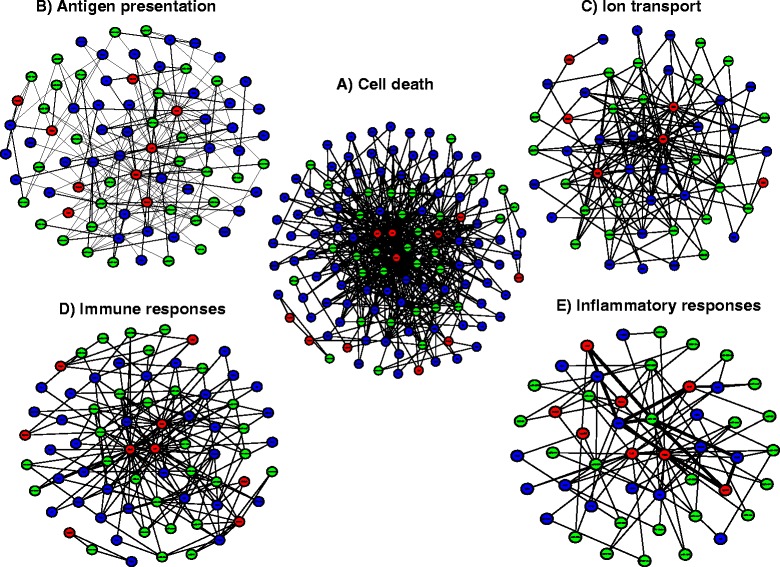
Sub-networks at 7d associated with 5 particular cellular processes all being a part of the larger regulatory network seen in Figure 3B. **A**. Loop-motifs related to cell death. **B**. Loop-motifs related to antigen presentation. **C**. Loop-motifs associated with ion transport. **D**. Loop-motifs associated with immune responses. **E**. Loop-motifs associated with inflammatory responses. The thicker edges highlight the loop motifs that involve rno-miR-185. This miR has been associated with inflammatory responses during brain ischemic stroke in mice and is potential target for prevention and treatment of stroke (ref. [65]). Green filled circles denote miRs, red filled circles denote TFs and blue filled circles denote target genes/transcripts.

There are 5 regulatory sub-networks, each corresponding to 5 categories of biological processes at the 7d time point as shown (Figure 6A-E). Together, these represented 61% of the total regulatory loop motifs observed at 7d. The numbers of regulatory loops for each process are listed (Table 2). In contrast to the 3 principal transcriptional regulators observed at 24 h (*Maf*, *Creb1* and *Stat1*), the major transcription factors *Stat1, Lef1* and *Bcl6* were present in all sub-networks linked to the 7d time point. The target gene-transcripts in each of the sub-networks were associated with the biological functions listed (Table 2). For example, the cell surface glycoprotein *Icam1*, endothelin (*Edn2*) and its receptor (*Ednrb*), the component of the innate immune system (*Cd14*), the activator of antigen presenting cells (*Cd40*) were among the hub genes in the antigen presentation associated sub-network (Figure 6B), while the gene transcripts for several subunits of potassium channels as well as for solute carrier membrane transporters were among the hubs located within the sub-network associated with ion transport (Figure 6C). The G protein-coupled receptor (*Hrh4*), as well as, Mediterranean fever (*Mefv*), the inducible heme oxygenase-1(*Hmox1*) and the Neutrophil cytosol factor 1 (*Ncf1*) were hubs in the network associated with inflammatory responses (Figure 6E).

**Figure 7:**
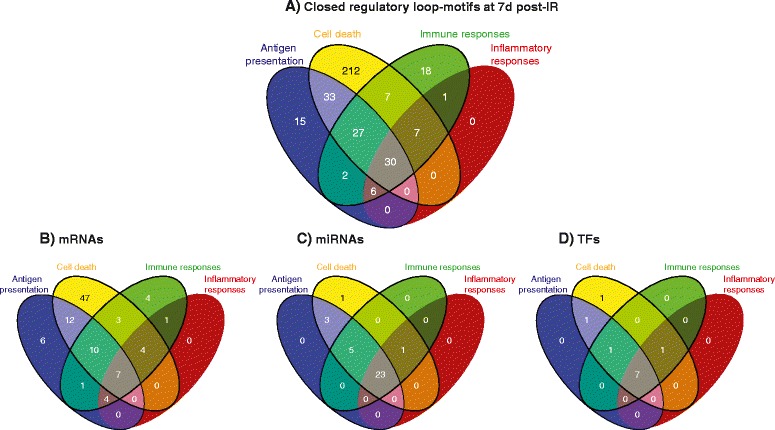
Venn diagrams representing 4 unique and overlapping cellular processes and molecular components at 7d. **A**. The numbers of common and unique loop-motifs associated with 4 cellular processes. **B**. The numbers of common and unique mRNAs (gene-transcripts) associated with 4 cellular processes. **C**. The numbers of common and unique miRNAs associated with 4 cellular processes. **D**. The numbers of common and unique TFs associated with 4 cellular processes. Colored oval shapes represent different biological processes. Blue: antigen presentation, yellow: cell death, green: immune response, red: inflammatory response.

There were 30 shared regulatory closed loops among the four sub-networks presented in Figure 7A. The ion transport associated network at the 7d time point was not included in this comparison. However, its similarity and differences to the ion transport process seen at 24 h are presented later. There were seven mRNA-transcripts common to the identified biological categories at 7d. In contrast, a large number of the miRs (23) and almost all TFs (12) were common to these biological categories (Figure 7B-D). Similar to our findings for the 24 h, the same TFs and miRNAs act through different regulatory loop motifs to regulate target gene-transcripts associated with different biological categories.

### Cell death sub-networks at 24 h vs. 7d post IR-injury

**Figure 8:**
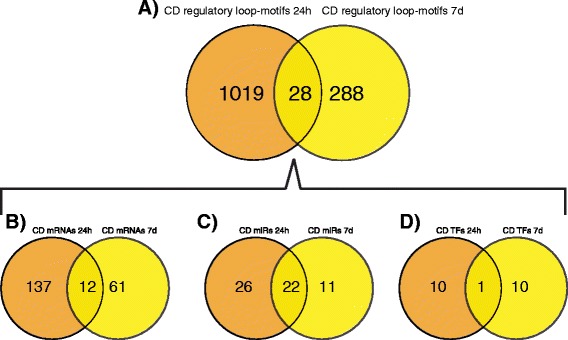
The numbers of unique and common loop-motifs and molecular components related to cell death at early (24 h) and late (7d) stages of ischemia-reperfusion injury in the retina. **A**. Unique and common loop-motifs associated with cell death at 24 h and 7d. **B**. Unique and common mRNA associated with cell death at 24 h and 7d. **C**. Unique and common microRNAs associated with cell death at 24 h and 7d. **D**. Unique and common transcription factors associated with cell death at 24 h and 7d. The pattern of node relationships for the molecular components is typical in that the numbers of TF < miRs < mRNAs.

We further looked at the regulation of retinal cell death at the two time points, 24 h and 7d, following IR-injury to see if the regulatory loop motifs are the same or not. The results from this analysis are summarized (Figure 8). Only 28 closed regulatory loop motifs at 24 h and 7d (representing 2.7% and 8.8% respectively) were common. The common motifs consisted of 12 mRNAs, 1 TF (*Stat*1) and 22 miRs. The numbers of time point specific target genes and TFs exceeded by far the number of the common ones (Figure 8B and D), which was less true for the miRs (Figure 8C). This result suggests that retinal cell death is a result of altered expression of different target genes in 24 h versus 7d post-IR time points and their transcription is regulated by different transcription factors. However, there are many common miRs that fine-tune the expression of diverse cell death related genes in 24 h and 7d post-IR stages.

### Ion transport sub-networks at 24 h vs. 7d post IR-injury

**Figure 9:**
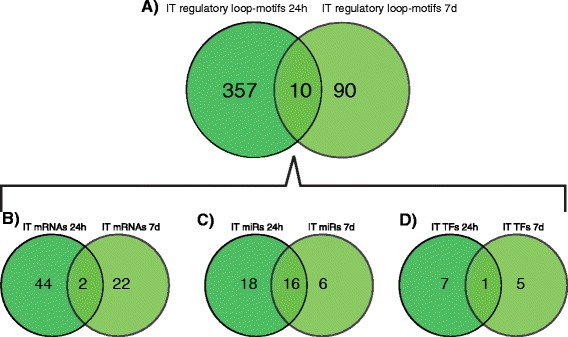
The numbers of unique and common loop-motifs and molecular components related to ion transport at early (24 h) and late (7d) stages of ischemia-reperfusion injury. **A**. Unique and common loop-motifs associated with ion transport at 24 h and 7d. **B**. Unique and common mRNAs associated with ion transport at 24 h and 7d. **C**. Unique and common microRNAs associated with ion transport at 24 h and 7d. **D**. Unique and common transcription factors associated with ion transport at 24 h and 7d. The pattern of node relationships for the molecular components is typical in that the numbers of TF < miRs < mRNAs.

We queried the data to determine if the same regulatory loops and their molecular components were involved in the regulation of ion transport at the 24 h and 7d time points. The results from this analysis are summarized (Figure 9). Only 10 closed loop motifs (representing 2.7% and 10% from the total ion transport associated motifs at 24 h and 7d, respectively) were found in the intersection between the ion transport processes at both post-ischemic periods. The common motifs consisted of 2 mRNAs (*Itpr*2 and *Kcnj*3), 1TF (*Stat*1) and 16 miRs. This pattern, like the pattern seen for cell death, indicates that the TFs and genes are mostly unique, whereas a larger percentage of the miRs are shared among the loops across the two time points.

## Discussion

We analyzed mRNA and miRNA arrays for ischemic-reperfusion injury in the rat retina for 0 h, 24 h and 7 days following a 1 h ischemic period. We developed a protocol to look at the correlated expressions between 3 nodes, miRs, mRNAs and TFs, connected by edges, in what we have termed closed loop-motifs. All three molecular elements are required to be related source/targets and have correlated expressions in order to be part of a closed loop. A context dependent and regulatory relationship between the 3 members of these loop-motifs is inferred. The edges in a closed loop-motif show significantly correlated regulation between the 3 nodes. Because of this particular requirement, our loops happened to contain only positive correlations. In this analysis, we have not given any weight to any particular direction of interaction. However, we made every attempt to show that the members of the closed regulatory loops are related, such that they have significantly correlated expressions and that the TF and the miR are known to interact with the target gene or its transcript in the closed regulatory loop. The 0 h time point showed few changes in correlated expression, none of which reached the level of statistical significance in our particular analysis. So, we focused our efforts on the 24 h post-IR and 7d post-IR time points, which will hereafter be referred to as “early” and “late” times respectively. Compared to our earlier study [56] we made significant improvements to our analytical approach, for example, we used a stringent filter to select differentially expressed IR-related molecular components (corrected p-value vs p-value). We also increased the numbers of TFs and their targets by using a promoter analysis with the aid of the TRANSFAC commercial database (instead of the publicly available TF-TG pairs used in our previous study). These improvements increased the number of the closed loop motifs from 87 to 4218 for the early post-IR time and from 140 to 957 for the late time point.

We showed that the regulatory networks associated with the early and late times post-IR injury shared only relatively few closed loop motifs, which indicated that there were mostly different sets of loop motifs involved in the two stages. This finding illustrates the potential of the loop motifs to be diagnostic markers in retinal pathologies. In addition, other recent studies have highlighted the significance and the possible applications of the regulatory loops in the predictive and preventive medicine [17,57-59]. We highlighted important regulatory elements, such as miRs and TFs, associated with early and late phases of post-IR injury periods. Some of the regulators seen here were previously associated with ischemic-related injury in other tissues, while others have not yet been linked to ischemia. For example, the top three miRNA-hubs in the early IR-injury regulatory network were rno-miR-495, rno-miR-214 and rno-miR-298, whereas rno-miR-873, rno-miR-223 and rno-miR-185 were hubs observed at the late phase post-IR injury. A recent study showed that inhibition of miR-495 increased neovascularization and recovery of blood flow after cardiac ischemia in mice [60], while miR-214 protected the mouse heart from ischemic injury by controlling Ca^2+^ overload and cell death [61] (Table 5). Up-regulation of miR-298 was previously reported in both, brain and blood, after ischemic stroke [62], while up-regulation of miR-873 was reported after onset of focal cerebral ischemia in mice [63]. It has been shown that miR-223 was neuroprotective by targeting glutamate receptors in mice brain, since overexpression of miR-223 decreased the levels of GluR2 and NR2B, inhibited NMDA-induced calcium influx in hippocampal neurons, and protected the brain from neuronal cell death following transient global ischemia and excitotoxic injury [64]. MiR-185 has been associated with inflammatory responses during brain ischemic stroke in mice and may provide underlying target for prevention and treatment of stroke [65]. We found that in the late phase of post-IR injury rno-miR-185 participates in four different loop-motifs (indicated by the thicker edges in Figure 6E) and these loop motifs are part of the sub-network linked to inflammatory responses.

**Table 5:** **Rat retinal miRs showing higher connectivity in the large gene networks associated with early and late post-IR injury periods along with their reported activities in other systems and relevant citations**

**miRNA**	**Time point**	**Regulation/function**	**Refs**
Rno-miR-495	Early (24 h)	inhibition of miR-495 increased neovascularization and recovery of blood flow after cardiac ischemia in mice	[60]
Rno-miR-214		miR-214 protected the mouse heart from ischemic injury by controlling Ca^2+^ overload and cell death	[61]
Rno-miR-298		miR-298 was up-regulated in brain and blood after ischemic stroke	[62]
Rno-miR-206		miR-206 was significantly deregulated during the conditions of unfolded protein response in H9c2 rat cardiomyoblasts	[79]
Rno-miR-221		miR-221 was suggested as a biomarker for cerebrovascular disease. Stroke patients and atherosclerosis subjects showed significantly lower miR-221 serum levels than healthy controls	[80]
Rno-miR-873	Late (7d)	miR-873 was up-regulated after onset of focal cerebral ischemia in mice	[63]
Rno-miR-223		miR-223 targeted glutamate receptors in mice brain. Overexpression of miR-223 decreased the levels of GluR2 and NR2B, inhibited NMDA-induced calcium influx in hippocampal neurons, and protected the brain from neuronal cell death following transient global ischemia and excitotoxic injury	[64]
Rno-miR-185		miR-185 has been associated with inflammatory responses during brain ischemic stroke in mice and may provide underlying target for prevention and treatment of stroke	[65]
Rno-miR-329		Inhibition of miR-329 increased neovascularization and blood flow recovery after ischemia in mice subjected to double femoral artery ligation	[60]
Rno-miR-138		hypoxia-induced miR-138 is an essential mediator of endothelial cell dysfunction via targeting S100A1 Ca^2+^ sensor	[81]

We showed that transcription factors, *Maf, Creb1* and *Stat1*, were the 3 principal hubs with high connectivity in the early phase IR-injury regulatory network, whereas *Stat1, Lef1* and *Bcl6* were the 3 principal hubs in the late phase IR-injury network. Each of these transcription factors is evidently a central coordinator because they regulate such large numbers of targets in the corresponding early and late phases of IR-injury. Reportedly, really important hubs tend to be shared by several tissues [66]. *Stat1* was the only common TF between the early and late post-IR times. *Stat1* has been identified as a hub gene in several tumor-associated transcriptional networks [67] including a gene network within HeLa cells exposed to IFNγ [68]. In addition, altered *Stat1* levels have been reported in rats with focal cerebral ischemia [69] but also in glaucomatous rat retinal ganglion cells [70]. The transcription factor *Bcl6* has been identified as an important hub in gene regulatory networks associated with eight human tissues [66] and its critical role in preventing apoptosis in the retina during early eye development was previously reported [71]. Increased phosphorylation of CREB in the brain was observed in two rat models of ischemic preconditioning [72] and *Creb*1 expression was detected in canine and human retinas affected with age-related macular degeneration (AMD) [73]. Maf members form a distinct family of the basic leucine zipper (bZip) transcription factors and have been involved in various disease pathologies (reviewed in Ref [74]). A member of the Maf family, the leucine zipper protein Nrl, is neural retina-specific and has been shown to regulate the expression of rod-specific genes, including rhodopsin [75]. Taken together, the six hub-TFs described above seem to regulate all biological processes in a particular phase following IR-injury, which indicated an important role for these regulators in the pathogenesis of this disorder in the retina.

Approximately 50% of the loop-motifs from the regulatory network associated with the early phase were involved in five cellular processes. All of these could ultimately lead to cell death, as indicated by the large number of loops shared with the cell death process. This implies that IR-injury initiates transcriptional and post-transcriptional regulatory interactions that contribute to neurodegeneration. The remaining loop-motifs were not associated with well-defined cellular processes. For comparison, 61% of the regulatory motifs from the network related to the late phase of IR-injury were linked to cellular processes, the largest of which were cell death, antigen presentation and immune responses. This indicates that the IR-injury related regulatory networks were consistent with previous studies in terms of affected cellular process [29,70,76-78]. However, the novel component in the present study is the integration of the regulatory elements, miRs and TFs, in closed loops, sub-networks and large regulatory networks that associated with particular biological processes during the early and late phases of ischemia-reperfusion injury in the retina.

## Conclusions

The etiology of retinal ischemia-reperfusion injury is orchestrated by complex and still not well understood gene networks. This work represents the first large network analysis to integrate miRNA and mRNA expression profiles in context of retinal ischemia. Importantly, we highlighted the regulatory elements of miRs and TFs within these gene networks, and found specific miRs and TFs, associated with biological processes in the early and late phases of ischemia-reperfusion injury. It is likely that an appreciation of such regulatory networks will have prognostic potential. In addition, the computational framework described in this study can be used to construct miRNA-TF interactive systems networks for various diseases/disorders of the retina and other tissues.

## Additional file

Additional file 1:
**Results from Pearson and distance correlations tests for the TF-Gene, miR-Gene and miR-TF pairs used to construct the regulatory loop motifs associated with early and late time points following retinal ischemia.**
